# Influence of Early versus Late supplemental ParenteraL Nutrition on long-term quality of life in ICU patients after gastrointestinal oncological surgery (hELPLiNe): study protocol for a randomized controlled trial

**DOI:** 10.1186/s13063-019-3796-3

**Published:** 2019-12-27

**Authors:** Paweł Piwowarczyk, Paweł Kutnik, Michał Borys, Elżbieta Rypulak, Beata Potręć-Studzińska, Justyna Sysiak-Sławecka, Tomasz Czarnik, Mirosław Czuczwar

**Affiliations:** 1 II Department of Anesthesiology and Intensive Care, Medical University of Lublin, ul. Staszica 16, 20-081 Lublin, Poland; 2 Student’s Scientific Association at II Department of Anesthesiology and Intensive Care, Medical University of Lublin, Lublin, Poland; 3 Department of Anesthesiology and Intensive Care, Opole University Hospital, Wincentego Witosa 26, 45-401 Opole, Poland

**Keywords:** Supplemental parenteral nutrition, cancer, gastrointestinal surgery, quality of life, protein, ICU

## Abstract

**Background:**

Nutrition plays a major role in intensive care unit (ICU) treatment, influencing ICU length of stay and patient’s survival. If preferable enteral nutrition administration is not feasible, ESPEN and ASPEN guidelines recommend initiation of a supplemental parenteral route between the first and seventh day, but exact timing remains elusive. While rapid development in critical care enabled significant reduction in the mortality rate of ICU patients, this improvement also tripled the number of patients going to rehabilitation. Thus, it is quality of life after ICU that has become the subject of interest of clinicians and healthcare policy-makers. A growing body of evidence indicates that protein turnover in the early phase of critical illness may play a crucial role in the preservation of lean body mass. A negative protein balance may lead to muscle wasting that persists weeks and months after ICU stay, resulting in deterioration of physical functioning. Folliwing oncological gastrointestinal tract surgery, patients are threatened with negative protein turnover due to cancer and extensive surgical insult.

**Methods:**

This is a multi-centre, single-blinded, randomised controlled trial. The study population includes patients admitted to ICU units after major oncological gastrointestinal surgery that require supplemental parenteral nutrition. After initiation of enteral nutrition, the intervention group receives remaining daily requirement via supplemental parenteral nutrition on the first day of ICU stay while the control group is not supplemented parenterally until the seventh day of ICU stay while enteral nutrition is gradually increased.

Primary endpoint: long-term quality of life measured in the physical component score (PCS) of SF-36 questionnaire at 3 and 6 months after ICU admission.

**Discussion:**

To our knowledge, this is the first trial to investigate the influence of early supplemental parenteral nutrition on long-term quality of life after major oncological gastrointestinal surgery. We assume that, particularly in this population of patients, early supplemental parenteral nutrition may increase the long-term quality of life. The study construction also allows establishment of patients’ PCS SF-36 score prior to surgery and mean change in PCS SF-36 score during the recovery period, which is rarely seen in studies on critically ill patients.

**Trial registration:**

ClinicalTrials.gov: NCT03699371 registered on 12 October 2018.

## Background

### Overview

Nutrition plays a major role in intensive care unit (ICU) treatment, influencing not only ICU length of stay and in-hospital mortality but also the patient’s long-term quality of life. According to the American Society of Parenteral and Enteral Nutrition (ASPEN) and the European Society of Parenteral and Enteral Nutrition (ESPEN) the enteral nutrition (EN) route is preferential to the parenteral nutrition (PN) route to provide the daily protein and energy requirements for ICU patients (1.2–2 g protein/kg/day; 20–25 kcal energy/kg/day). However, in many cases, this cannot be met exclusively via EN due to a multitude of reasons. ASPEN and ESPEN guidelines then recommend the use of supplemental parenteral nutrition (SPN). Optimal timing of SPN remains elusive in the acute phase of critical illness (between first and seventh day after admission to ICU). A growing body of evidence underlines the crucial role of protein turnover in ICU patients [1].

### Existing knowledge

The importance of nutritional support is well established in the existing literature. Wischmayer et al. [2] raise awareness of the impact of interventions during ICU stay on long-term outcomes. The authors underline that, although rapid development in critical care enables a significant reduction in mortality rate, at the same time, it resulted in tripling the number of patients undergoing rehabilitation. Moreover, recent studies have proven that loss of lean body mass can reach a kilogram a day in critically ill or post-surgical patients [3]; most of this weight loss takes place during the first 7 days of ICU stay. However, the prevalence of fat tissue in the mass gained by patient following this may influence the future physical functioning after critical illness. Additionally, there are reports associating impaired wound healing, spontaneous wound development or even increased mortality secondary to pneumonia with extensive lean body mass loss [4]. It is worth noting that a catabolic/hypermetabolic state can persist for up to 2 years from hospital discharge and, although patients usually gain weight back after the ICU discharge, most of this weight is just fat mass.

Lately, the evidence on the role of optimal provision of protein in the prevention of muscle wasting is accumulating. Shifting the protein balance from negative to positive during the early stage of admission to ICU was proven possible by infusion of high level of amino acids (1 g/kg/day) [5]. Furthermore, the extent of the effect of the amino acid bolus, which was observed at the end of 3-h infusion, is present at 24 h [6]. Thus, these studies advocate that protein turnover remains stable even during critical illness. Moreover, studies on other populations (e.g. elderly, athletes) have proven that protein synthesis can be stimulated by bolus infusions [7, 8].

### Aim of the study

Our goal is to compare the influence of early and late SPN on the long-term quality of life (measured by the 36-Item Short Form Health Survey (SF-36) scoring system) in a population of critically ill patients after oncological surgery of the gastrointestinal tract at 3 and 6 months after admission to ICU.

### Hypothesis

We hypothesise that early goal-directed supplementation of proteins in SPN may significantly increase the number of points in the physical component score (PCS) at 3 and 6 months after admission to ICU in the population of critically ill patients after oncological surgery of the gastrointestinal tract.

### The need for the trial

Little is known about the effects of continuous low-dose protein administration on lean body mass in the early phase of critical illness. There are only a few studies on the impact of early protein delivery on long-term physical quality of life after ICU [9], whereas no studies assess this issue in a population of patients after oncological surgery of the gastrointestinal tract, who are highly threatened with a persistent catabolic state following the mixed effect of carcinogenesis and surgical insult. The potential benefits of this trial include not only the identification of factors that could alter the rate of individual physical impairments but it could also address the issue of growing costs of rehabilitation of ICU survivors to the healthcare system.

## Methods/design

### Study setting

The study will be conducted at the II Department of Anesthesiology and Intensive Care at the First Teaching Hospital in Lublin (Medical University of Lublin) and Department of Anesthesiology and Intensive Care at University Hospital of Opole (Medical University of Opole), Poland.

### Objective

This randomised trial will investigate the effect of an early supplemental nutrition therapy during intensive care, on long-term physical quality of life and short-term clinical outcomes.

### Trial design

Multi-centre, single-blinded, randomised controlled trial.

### Trial population

The trial population will include patients admitted to the ICU after major gastrointestinal surgery due to cancer who require SPN.

### Interventions

#### Intervention group

The intervention group will receive EN reaching up to 20% of daily nutritional requirements and early (on day 1 of their ICU stay) provision of up to 80% of their protein (2 g/kg/day or, in case of continuous renal replacement therapy (CRRT), 2.5 g/kg/day) and caloric (15–20 kcal/kg/day) needs in SPN, which will be continued until day 7 of ICU stay for the purpose of the study.

#### Control group

The control group will receive EN reaching up to 20% of their daily nutritional requirements and late provision (of up to 80%) of their protein (2 g/kg/day or, in case of CRRT, 2.5 g/kg/day) and caloric (15–20 kcal/kg/day) needs in SPN on day 7 of ICU stay if it is not already met via EN.

#### Route of administration

SPN is continuously infused for 24 h via central venous catheter. The central venous catheter is placed for routine medical purposes and therefore is not a part of any study-related procedure.

#### Dosage

SPN will be administered in a volume that provides up to 80% of the targeted proteins and calories. If energy is provided from other sources (e.g. via supplemental EN/oral nutrition or non- nutritional sources such as glucose solution for drug dilution or lipids from propofol), the dose of the SPN will be reduced accordingly. For dose calculation, the patient’s estimated body weight at the time of admission to the ICU will be used.

#### Administering of nutrition

The ICU nurses will administer the PN or EN according to the allocation. The SPN solutions will be prepared in a hospital pharmacy based on the ICU specialist’s prescription. The EN solutions are ready-to-use products prepared by manufacturers, details of which will be provided in the study report. Any problem related to nutrition will be reported by ICU nurses to ICU specialists. The site Principal Investigator is responsible for the adherence of administration of nutrition to the study protocol. Full and empty parenteral solution bags will be counted daily.

#### Concomitant care

The implementation of either the intervention or control group protocols will not require alteration to other usual care pathways. Relevant concomitant care will be recorded according to the study protocol.

### Outcomes

#### Primary endpoint

The long-term quality of life will be measured by the physical component of the 36-SF questionnaire assessed by a phone call at 3 and 6 months after admission to the ICU (License number QM047431 CT198126 OP073545 OGSR).

#### Secondary endpoints

A. During the 7-day treatment period in both treatment arms:
  1. EN route intolerance (inability to administer up to 60% of protein needs on day 3 via EN route)
  2. Change from baseline in ultrasound measured thickness of diaphragm on days 1, 3 and 5 of ICU stay
  3. Protein delivery
  4. Energy intake
  5. Insulin dose
  6. Blood glucose profile
  7. Organic phosphorus level
B. During the 28-day treatment period in both treatment arms:
  8. Change from baseline in the Sequential Organ Failure Assessment (SOFA) score
  9. Duration of mechanical ventilation
  10. Length of stay in the ICU
  11. ICU mortality
  12. Length of stay in hospital
  13. Hospital mortality
  14. New onset of hospital-acquired infection (defined according to the Centers for Disease Control and Prevention surveillance definition as a localised or systemic condition resulting from an adverse reaction to the presence of an infectious agent(s) or its toxin(s), with no evidence that the infection was present or incubating at the time of admission to the acute care setting)
  15. Antibiotic-free days

### End of trial

Recruitment will cease when the last patient is randomised following our sample size recommendation. The end of the trial is expected when the last 6-month follow-up data is collected.

### Sample size

Target sample size: 220 (110 in each intervention and control group).

### Rationale

According to the data presented by Allingstrup et al. [9] we estimated that 200 patients are needed to show a 15% relative reduction in the primary outcome (physical component of SF-36 score at 6 months) corresponding to a difference of 5.5 points (minimal clinical important difference defined as half a standard deviation from the observed dataset in the presented study) between the intervention and the control group at a significance level of 0.05 and a power of 80%. The calculations were based on a PCS of 37.5 (SD 10.65) among survivors from their data. The PCS SF-36 did not differ between the intervention and control groups at 6 months in the general ICU population in this study. Moreover, we increased the number of patients in our study to 220 assuming a 10% loss to follow-up among survivors from ICU. We hypothesised that early SPN might influence the long-term outcome of critically ill patients after oncological surgery of the gastrointestinal tract that constituted only 6% of the intervention group and 12% of the control group of the study mentioned above.

### Recruitment

Patients enrolled in the study will be recruited from the population scheduled for gastrointestinal cancer surgery at the II Department of General and Gastrointestinal Surgery and Surgical Oncology of the Alimentary Tract and at Department of Surgical Oncology at First Teaching Hospital in Lublin, Poland, and the Department of General and Vascular Surgery at University Hospital in Opole, Poland.

## Methods

### Assignment of intervention

#### Enrolment procedure

**Fig. 1:**
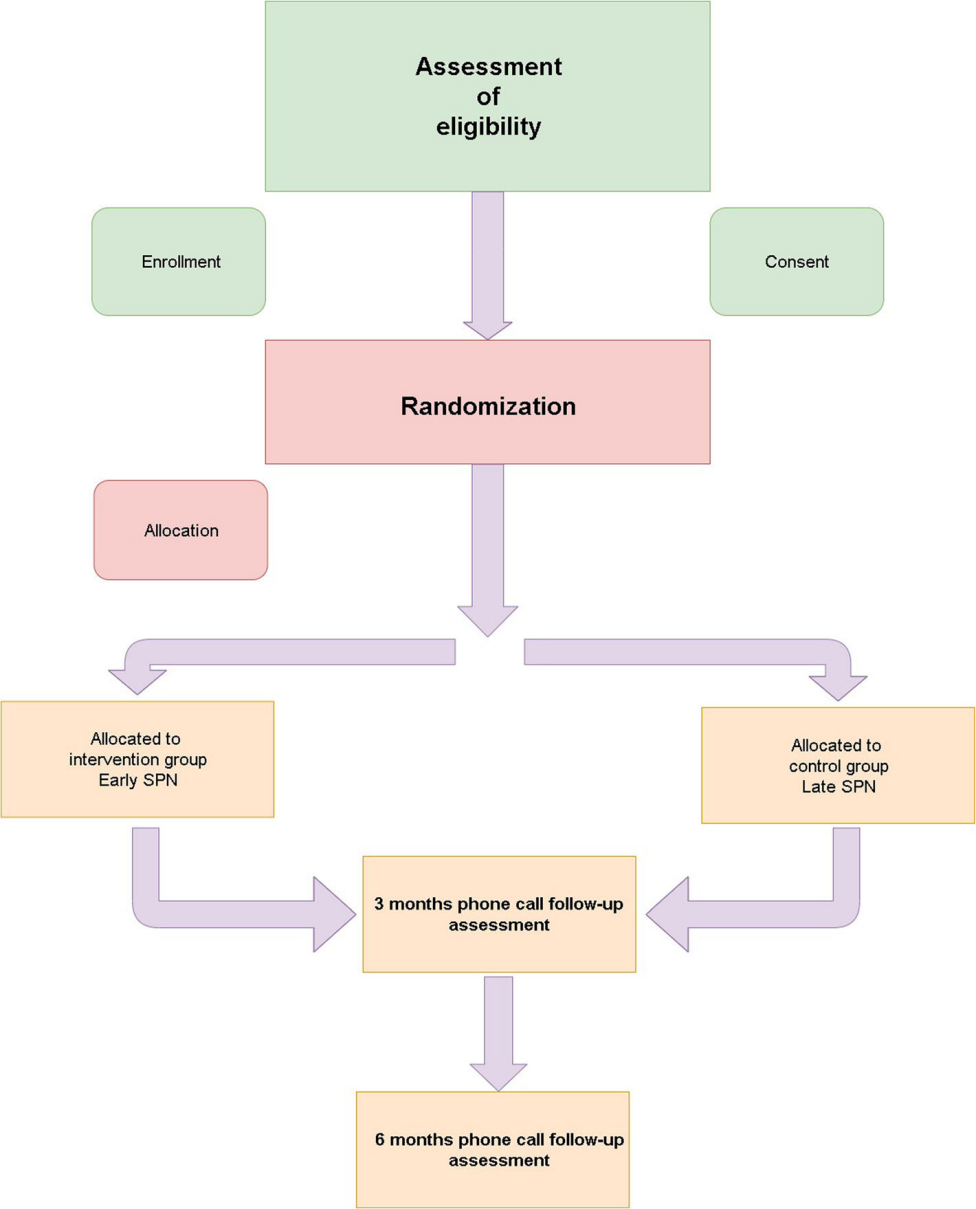
Chart flow

All study participants will be enrolled from surgical departments by anaesthetic residents during presurgical assessment according to the inclusion and exclusion criteria (Table 1 and Fig. 1). Prior to entry, the patient, next of kin or legal representatives will need to sign the consent forms.

**Table 1:** Eligibility criteria

Inclusion criteria	Exclusion criteria	
1. ICU patients in the acute phase of critical illness after gastrointestinal oncological surgery 2. Age ≥18 years 3. Central venous access available for continuous infusion of the study drugs 4. Admitted to the ICU during the previous 24 h with a minimum expected ICU stay of ≥5 days 5. Sequential Organ Failure Assessment (SOFA) score ≥ 2 6. Written informed consent from the patient or the patient’s legal representative	Exclusions associated with nutritional status: 1. Received parenteral nutrition within 7 days before randomisation 2. Expected to receive ≥20% of energy via supplemental EN and/or non-nutritional sources (e.g. glucose solution for drug dilution or lipids from propofol) during the first 3 nutritional treatment days 3. Inability to initiate EN prior to randomization 4. Body mass index <17 kg/m^2^ or >35 kg/m^2^ 5. Any congenital errors of amino acid metabolism Exclusions associated with comorbidities and allergies: 6. Known hypersensitivity to fish, egg, soybean proteins, peanut proteins, or to any of the active substances or excipients contained in SPN 7. Known hypersensitivity to milk protein or to any other substance contained in SPN 8. Hemophagocytic syndrome 9. Known history of HIV, hepatitis B and/or C 10. Any severe, persistent blood coagulation disorder with uncontrolled bleeding Concomitant therapy exclusions: 11. Chronic maintenance therapy with systemic glucocorticoid steroids (hydrocortisone >0.3 mg/kg/d). 12. Concomitant administration of chemotherapy 13. Administration of growth hormone and teduglutide within the previous 4 weeks	Laboratory exclusions: 14. Hypertriglyceridemia characterised by serum triglyceride levels >4 mmol/L (>350 mg/dL) 15. Treatment-refractory, clinically significant major abnormality in the serum concentration of any electrolyte (sodium, potassium, magnesium, total calcium, chloride, inorganic phosphate) 16. Acute liver failure with encephalopathy, including intoxication (e.g. paracetamol, death cap, golden chain) and/or liver enzymes (aspartate aminotransferase, alanine aminotransferase, gamma glutamyl transferase) or bilirubin exceeding 10 x upper limit normal Other exclusions: 17. Chronic liver failure (Child-Pugh scale B or C), e.g. secondary to drug or alcohol abuse 18. Participation in another interventional clinical trial within the previous 4 weeks 19. Pregnancy or lactation 20. Previous inclusion in the present study 21. Patient unlikely to survive to 6 months due to underlying illness 22. Receiving end-of-life-care

*EN* enteral nutrition, *ICU* intensive care unit, *SPN* supplemental parenteral nutrition

#### Randomisation

Each eligible patient is allocated randomly during admission to one of the two groups: the early SPN or the late SPN group. The randomisation process is based on the order of admission to ICU, during which each eligible patient is given a consecutive number. Subsequently, a sealed envelope with a matching number is opened, which contains the name of either the intervention or the control group. The envelopes will be concealed and prepared according to the 1:1 ratio computer-generated randomisation table by a medical secretary who will not be involved in the trial.

#### Allocation concealment

Patients will be allocated to the early SPN or the late SPN group according to the given randomisation stored in the closed envelopes. The table will be stored in a designated safe by the medical secretary not involved in the study.

#### Equipment used

In order to assess thickness of the diaphragm on days 1, 3 and 5 of ICU stay, a Philips CX50 Portable Ultrasound Machine and a Philips Sparq Ultrasound Machine will be used.

### Participant data collection

Participant background data of age, sex, medical history, primary diagnosis, weight, height, body mass index and baseline PCS SF-36 will be collected.

#### Clinical testing data

- Prior to enrolment, haemoglobin, platelet, Na^+^, K^+^, Ca, Cl^–^, Mg^2+^, P, triglyceride, alkaline phosphatase, aspartate aminotransferase, alanine aminotransferase, gamma glutamyl transferase and bilirubin levels, International Normalised Ratio, and activated partial thromboplastin time as well as presence of HIV, hepatitis B virus or hepatitis C virus will be assessed.
- Daily from days 1 to 7, haemoglobin, leukocyte, platelet, lactic acid, bicarbonate, urea, creatinine, bilirubin, alanine aminotransferase, aspartate aminotransferase, gamma glutamyl transferase, alkaline phosphatase, glucose, phosphorus, Na^+^, K^+^, Ca^2+^, Cl^–^, Mg^2+^, C-reactive protein, and procalcitonin levels as well as peripheral capillary oxygen saturation (SpO_2_), the arterial oxygen partial pressure to fractional inspired oxygen ratio (PaO_2_/FiO_2_), blood pH, partial pressure of carbon dioxide in arterial blood (PaCO_2_) and fluid balance will be measured.
- Daily from days 1 to 28 (or until discharge), the SOFA score will be measured.
- On days 1 and 7 of ICU stay, albumin and total proteins will be measured.
- On days 1, 3 and 5 of ICU stay, ultrasonographic assessment of the thickness of diaphragm will be performed.

#### Treatment and support in-hospital data

All performed surgeries will be laparoscopic or opened abdominal/thoracic surgery. The surgical sites will be grouped into oesophageal procedures, gastric procedures, bile duct, liver or pancreatic procedures, small bowel procedures, colonic procedures, and rectal procedures.

Postoperative surgical course will be assessed for postoperative complications according to the 7-grade Clavien–Dindo classification.

The level of organ support and pharmacotherapy required will be assessed according to duration of CRRT and mechanical ventilation, antibiotic administration, gastric antisecretory agents, prokinetic agents, catecholamines and insulin dose (per 24 h).

The name of EN or PN, the target volume, received volume, energy intake, protein intake, intolerance to EN or PN support, and the decrease or discontinuation of nutritional support will be assessed.

The use of invasive devices such as central catheters, urinary catheters, enteral route devices and endotracheal tubes, with the dates of insertion and removal, will be recorded. All those parameters, treatments and devices will be controlled until day 28 or until discontinuation, whichever occurs first.

The occurrence of hospital-acquired infections such as ventilator-associated pneumonia, bacteraemia, catheter-related infections or urinary tract infections will be recorded.

#### Follow-up data

**Fig. 2:**
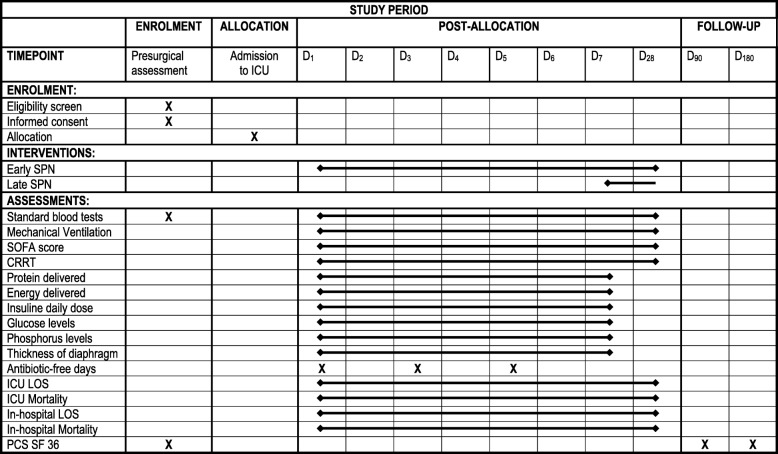
Study period

Each patient will be followed-up until day 180. The vital status will be recorded at ICU discharge, hospital discharge and on day 28. Follow-up data will be collected by study investigators and study nurses. We plan to collect two phone numbers per patient to increase the likelihood of patient follow-up at 3 and 6 months after allocation to the study group (Fig. 2).

### Statistical analysis

The assumption of this trial is to match the results in adherence to intention-to-treat analysis. Patient demographic data and baseline characteristics will be presented with the use of descriptive statistics. For the summary of continuous data, we plan to apply minimum and maximum values, medians, means, standard deviations and quartiles. A summary of categorical variables will be presented as distribution (with the use of percentage and numbers). We plan to analyse the primary outcome, which is the PCS SF-36, by Wilcoxon’s test and general linear regression. We expect data to be non-normally distributed.

For the secondary outcomes, we plan to use the χ^2^ test for dichotomous variables and Wilcoxon’s test as well as regression analyses for ordinal data. Time-to-event data will be compared with log rank test.

### Missing data

We assumed a 10% loss to follow-up among the population that completed the trial and enlarged the sample size from 200 to 220 participants.

### Blinding

Assessors will be blinded. However, the blinding of investigators and participants (masking of intervention) to the use of PN is not feasible. We do not anticipate any requirement for unblinding but, if required, investigators will have access to group allocations and any unblinding will be reported.

### Post-trial care

Conventional intensive care therapy will be continued according to the discretion of the intensivist.

### Harms and benefits

#### Potential burdens and risks, expected benefits

Potential adverse events and risks related to PN include, but are not limited to, infections (e.g. catheter-related infections), hyperglycaemia, refeeding syndrome, diarrhoea and liver dysfunction (e.g. bile sludge and cholestasis). The study protocol is designed in compliance with the present recommendations of ASPEN and ESPEN on optimal nutrition care in patients after surgery. The strategies used in both arms are classified as standard care; therefore, for example, adverse events associated with routine medical invasive procedures, such as central venous catheter placement, will not be treated as any study-related risks. There will be no special criteria for discontinuing or modifying allocated interventions. There is no anticipated harm and compensation to those who suffer harm from trial participation. The benefits of SPN for patient recovery are well documented. Complementary to previous studies, the results of this trial might elucidate the optimal timing of SPN in patients recovering from oncological gastrointestinal tract surgery in the long-term setting.

### Evaluations and reporting of adverse event

If any adverse event or serious adverse event occurs, the Principal Investigator will be informed immediately. The duty of the Principal Investigator is to record these in the patient’s requisition form and inform the Local Ethical Committee in order to establish the causal relationship between the event and trial interventions. Adverse events will be reported in the study publication.

### Monitoring

Independent monitoring of adherence of the investigators to Good Clinical Practice regulations and of the data collection process will be held every 6 months. The Data Monitoring and Ethics Committee will review confidential interim analyses of accumulating data annually.

### Confidentiality

#### Data management

All actions will be undertaken to ensure anonymity of the trial participants. Patient data will be collected on paper sheets and later uploaded to the study database, which will not contain identifiable personal information. Access to the study password-protected database will be granted exclusively to the investigators and study statisticians. Storage of any study-related information on portable memory devices will be minimised. Collected paper data will be stored in a designated archive. Printed and electronic data obtained for study purposes will be kept for 5 years from the time of publication and later disposed of. Polish and EU legal regulations regarding medical data storage will be followed. The procedure of data management will be audited by the Principal Investigator.

#### Protocol amendments

Changes to the study protocol will have to be accepted by the Local Ethical Committees of the participating centers. Recruitment of the study participants will begin only when the final study protocol is assessed and approved Additional file 1.

#### Dissemination policy

We plan to share the results of our study with the scientific and clinical community by reporting it at conferences and by submission to a scientific journal for publication.

## Discussion

Survival of critical illness and ICU mortality have been reduced in the last 10 years; however, the number of patients going to rehabilitation after ICU stay has tripled [10].

The factors that may influence the deterioration of physical quality of life after ICU need to be identified. A population particularly vulnerable to impairment in functioning after ICU stay are patients following expanded surgery of the gastrointestinal tract. Rapid development of national preventive and screening programmes have enabled early identification of resective gastrointestinal tumors, increasing the number of patients scheduled for that type of surgery. Nutritional support plays a major role in the recovery after extensive resections of the gastrointestinal tract. ESPEN and ASPEN recommend initiation of feeding via the EN route. However, early meeting of full nutritional requirements is rarely viable via the EN route alone, while adequate delivery of proteins might prevent the cascade of catabolism during the first days of ICU stay after extended surgery. Extensive muscle wasting may be a consequence of prolonged catabolic state. SPN is recommended when EN is not able to fulfill the daily nutritional requirements of those patients. Nevertheless, the optimal timing for introduction of SPN remains elusive. ESPEN guidelines suggest the introduction of SPN in first 7 days, while ASPEN recommends waiting until day 8. The a previous large trial, EPaNIC-NEJM, showed higher risks of adverse events after early introduction of SPN versus late introduction. It also showed no influence on mortality rate in either group. However, the issue of long-term quality of life was not covered discussed. The EAT-ICU trial [9], which was the only study investigating physical quality of life of the general population of patients after ICU, showed no improvement in PCS SF-36 in the group where early goal-directed nutrition was implemented. We hypothesize that early initiation of full nutritional support could significantly influence the physical quality of life of patients after oncological surgery of the gastrointestinal tract, a group of patients who are particularly vulnerable to the mixed catabolic effect of cancer and extensive surgery. Postsurgical patients admitted to the ICU constituted only 6% of the population studied in the EAT-ICU trial.

This is a multi-center, single-blinded, prospective randomised study designed to establish the influence of early SPN on the long-term quality of life of patients admitted to the ICU after oncological surgery of the gastrointestinal tract. The potential benefits of this trial would include not only identification of factors that could alter the rate of individual physical impairments but, moreover, it might address the issue of growing costs of rehabilitation of ICU survivors to the healthcare system. Potential limitations of this study would include lack of masking of the assigned intervention. Another drawback of this study protocol includes the fact that a proportion of patients after major gastrointestinal oncological surgery is not routinely admitted to the ICU. Thus, the results of the study may have limited generalisability to patients admitted postoperatively to the ICU. We addressed this limitation by identifying the proportion between the whole cohort of patients who underwent major gastrointestinal oncological surgery to those included in the trial. We set the follow-up period at 3 and 6 months due to the fact that the definitive answer to the question about physical functioning after ICU can be established only after returning to the original home setting, while hospitalisation of cancer patients is prolonged on many occasions Additional file 1.

## Supplementary information

**Additional file 1.** SPIRIT 2013 Checklist: Recommended items to address in a clinical trial protocol and related documents.

## Abbreviations

ASPEN: American Society of Enteral and Parenteral Nutrition
CRRT: continuous renal replacement therapy
EN: enteral nutrition
ESPEN: European Society of Enteral and Parenteral Nutrition
ICU: intensive care unit
PCS: Physical Component Score
PN: parenteral nutrition
SF-36: 36-Item Short Form Health Survey
SOFA: Sequential Organ Failure Assessment
SPN: supplemental parenteral nutrition
